# Global Patterns of City Size Distributions and Their Fundamental Drivers

**DOI:** 10.1371/journal.pone.0000934

**Published:** 2007-09-26

**Authors:** Ethan H. Decker, Andrew J. Kerkhoff, Melanie E. Moses

**Affiliations:** 1 Department of Biology, University of New Mexico, Albuquerque, New Mexico, United States of America; 2 Department of Biology and Department of Mathematics, Kenyon College, Gambier, Ohio, United States of America; 3 Department of Computer Science, University of New Mexico, Albuquerque, New Mexico, United States of America; Centre National de la Recherche Scientifique, France

## Abstract

Urban areas and their voracious appetites are increasingly dominating the flows of energy and materials around the globe. Understanding the size distribution and dynamics of urban areas is vital if we are to manage their growth and mitigate their negative impacts on global ecosystems. For over 50 years, city size distributions have been assumed to universally follow a power function, and many theories have been put forth to explain what has become known as Zipf's law (the instance where the exponent of the power function equals unity). Most previous studies, however, only include the largest cities that comprise the tail of the distribution. Here we show that national, regional and continental city size distributions, whether based on census data or inferred from cluster areas of remotely-sensed nighttime lights, are in fact lognormally distributed through the majority of cities and only approach power functions for the largest cities in the distribution tails. To explore generating processes, we use a simple model incorporating only two basic human dynamics, migration and reproduction, that nonetheless generates distributions very similar to those found empirically. Our results suggest that macroscopic patterns of human settlements may be far more constrained by fundamental ecological principles than more fine-scale socioeconomic factors.

## Introduction

Humans increasingly dominate the ecology and energy flows of the entire earth, prompting grave concerns about human population growth. However, the human population has not only doubled in the past 40 years, but that population is increasingly clustered in urban areas. In 1950, only 30% of the world's population lived in urban areas. By 2000 that proportion rose to 47%, and by 2030 that number will be 60%[1]. In fact, virtually all of the global population growth in the next 25 years will be urban, either through migration from rural areas, growth of existing cities, or the emergence of new urban clusters. In less developed countries, cities are burdened by the growth of unregulated slums, illegal or unmanaged waste and sewage disposal, and woefully inadequate water supplies, housing, and transportation infrastructure [2]–[4]. In more developed regions, rapid urban sprawl and the growth of the built-up urban fringe have outpaced much of the environmental and urban planning that attempt to manage them [3]–[6].

The increasingly global ramifications of human urbanization necessitate a global perspective on the problem. If we hope to successfully manage urban environmental impacts, we first need to know how urban areas are distributed and how that distribution varies around the world. Second, we need to understand what basic ecological principles (if any) underlie that distribution and how those principles embody themselves in human behavior. Finally, we need to understand how the per capita environmental impact of humans varies across settlements of different sizes and across regions that differ economically, culturally, and biogeographically. This paper addresses the first two points by quantifying the size distribution of urban areas around the world and modeling their ecological bases in the dynamics of human migration and reproduction.

Urbanization is occurring so quickly in many areas that it has become difficult to distinguish city, suburb, and town. There are many ways to define a city, e.g., as an incorporated area, an urban agglomeration, or a settlement with population density larger than some threshold value. All definitions have shortcomings: is Newark part of the New York City metropolitan area? Is Santa Fe, New Mexico, a city or a town? What about an urban area that straddles a county, state, or even national border, such as Kansas City or El Paso–Ciudad Juarez? For this paper we use the term city very loosely to mean any human settlement that is functionally coherent and denser than its surroundings, and we use it interchangeably with the term settlement. We do not distinguish here between villages, towns, cities, metropolitan areas, and megacities (though differences of kind certainly exist). Generally, we are interested in understanding the flow of energy and materials through human networks, and as virtually all commerce requires some aggregation of population to occur, we are interested in the entire set of human settlements.

### Current theories of city size distributions

Consider a set of cities from a region, such as a country, ranked by population (or by area) from largest to smallest. When population is graphed against rank, the shape of the curve describes the relative proportions of smaller and larger cities. In 1949, Zipf observed that the population of a city is proportional to the inverse of its regional rank [7], resulting in a power law that has an exponent of approximately −1. Equivalently, this observation, which has become known as “Zipf's law,” states that the probability that the size of a city *s* is greater than some *S* is proportional to 1/*S*: *P*(*s*>*S*) = *cS ^x^*. with *x* = −1.

The mechanisms underlying Zipf's law have been the subject of much theoretical debate [8]–[12]. Because pattern and process are intricately linked in natural phenomena [13]–[15], governing processes are often inferred from the observed patterns [16]; power laws are often taken as evidence that these processes are scale-invariant [17], [18]. Several researchers have hypothesized that Zipf's law is a result of all cities growing at the same rate, regardless of their size. This law of proportionate effect is also known as Gibrat's law [19]. Others suggest that a steady rate of new cities joining an urban system (i.e., Yule's theorem) produces the power function [8]. In fact, there is a wide variety of theories for Zipf's law, ranging from the statistical-mechanical to the sociological and political (discussed in Andersson 2002 [20], and Ioannides&Gabaix [21]). A few regional studies have suggested that a lognormal best describes the city size distribution [21], particularly when the smallest cities are included [22], [23]. Carroll [24] reviews much of the early literature on Zipf's law.

What almost all explanations have in common is the assumption that Zipf's law is a robust empirical pattern that requires explanation. However, we argue that insufficient consideration has been given to 1) testing the applicability of Zipf's law over the entire range of human settlement sizes, and 2) developing useful “neutral models” for understanding the extent to which settlement distributions represent stochastic vs. deterministic, goal-directed (e.g., optimization) processes. These two points are critical to assessing the generality and meaningfulness of Zipf's law, i.e., whether it actually teaches us anything about human ecology and the organization of human populations. Even on a more practical level, we cannot apply Zipf's law as even an empirical descriptor of the distribution of human settlements without more fully addressing its generality.

## Methods

### Empirical settlement size distributions

Most studies of city size distributions have concentrated on only the largest cities and have ignored smaller cities, towns, and settlements, mainly because suitably accurate data for small cities did not exist. Yet as much as 70% of the population may reside in these smaller areas; omitting that mass of the population may lead to biased characterizations of city size distributions.

**Figure 1:**
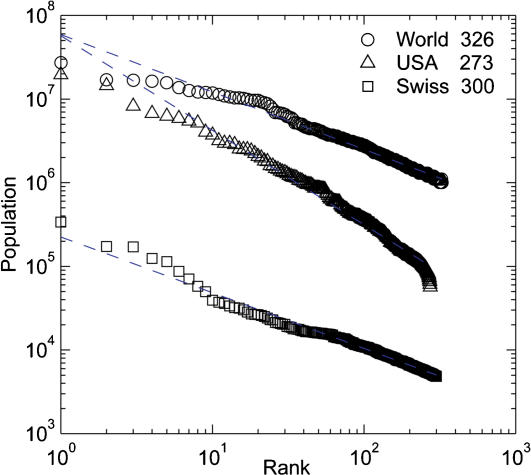
Rank-size distributions of large cities. Key indicates sample sizes. All three samples are fit well by power laws over two orders of magnitude in population, though all three exponents are significantly different from −1.

For many regions around the globe, large cities do follow power functions. Figure 1 shows the rank-size distributions and power-law fits of population *P* to rank *R* for the largest cities from three data sets: metropolises of the world [25] (*P* = 5.9×10^7^ *R* ^−0.686^, standard error of exponent = 0.006, *r* ^2^ = 0.847, *p*<0.00001), metropolitan areas of the USA [26] (*P* = 5.7×10^7^ *R* ^−1.129^, *s.e.* = 0.010, *r* ^2^ = 0.891, *p*<0.00001), and Swiss municipalities [27] (*P* = 2.3×10^5^ *R* ^−0.666^, *s.e.* = 0.005, *r* ^2^ = 0.960, *p*<0.00001). All three samples are fit well by power laws over two orders of magnitude in population, though all three exponents are significantly different from −1. (Population data used in this study are provided in Figure S1.)

**Figure 2:**
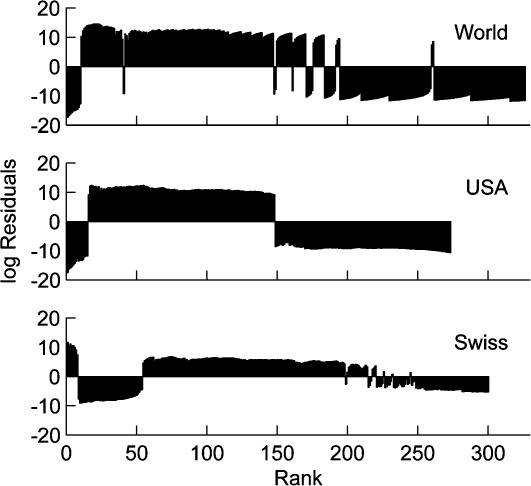
Logarithm of the residuals of cities from power law fits. Data from Figure 1. Residuals from a good model should be randomly distributed around zero. All three regions show systematic deviations from predicted values, indicating that city size patterns are not entirely described by power laws.

However, inspection of residuals for empirical data reveals systematic deviations from the scaling fit (Figure 2). Residuals from a good model should be randomly distributed around zero. All three regions show systematic deviations from predicted values, indicating that city size patterns are not entirely described by power laws.

Thus, while it has become a given that city size follows Zipf's law, this assumption has rarely been tested using truly comprehensive data. Here we will test the generality of Zipf's law across the entire range of city sizes, using more accurate and comprehensive population and nighttime light data. Population data for the United States [28] cover all areas designated ‘populated places’ by the U.S. Geological Survey, accounting for 178.6 million people or 65.2% of the nation. Census data for Switzerland [27] and the world [29] come from space-filling polygons, accounting for 100% of each population.

Unfortunately, polygon data are rarely coincident with actual city areas (e.g., a Swiss municipality could comprise part of a metropolitan area or several small towns), and census methods and reliability vary widely across regions around the world. Consequently, we also used the Nighttime Lights of the World data from the U.S. Defense Meteorological Satellite Program, Operational Linescan System (DMSP-OLS) [30]. By measuring faint, visible-near infrared emissions at the Earth's surface at night, the DMSP-OLS can detect cities, towns, and villages. The National Geophysical Data Center has produced georeferenced nighttime light maps with a 1-km^2^ resolution for major regions of the world using data recorded between October 1994 and March 1995. Only lights that are stable across several nights are classified as human settlements. This removes shipping&fishing vessels, wildfires, and other ephemeral or moving light sources from the dataset.

Nighttime light cluster area (in km^2^) correlates very well with population: for the United States, the area of DMSP-OLS light clusters predicts population with an *r* ^2^ between 0.63 and 0.93 depending on how the data are transformed [31]. Although the degree of correlation between population and night lights will vary globally, this analysis provides a single, consistent metric of size for the full range of human settlements around the world. Most previous studies were either regional in extent or relied on arbitrary definitions of cities that varied across regions. We also examined cluster area distributions for 11 continental and subcontinental regions of the world, which excludes only small islands, Antarctica and the Arctic.

### Model of urban dynamics

To generate “neutral” baseline expectations for how human settlement sizes should be distributed, we adapt a simple model of urbanization that Manrubia and Zanette used to study city size distributions [32]. Their results were consistent with their empirical studies of large cities and supported Zipf's law. However, as with their empirical work, they exclude small settlements. We modify their model to include a reproduction component (population growth) and replicate their study to examine whether Zipf's law indeed holds across the entire range of settlement sizes.

We make the simplest assumptions about migration and reproduction. We assume that on a global scale, human migration is essentially random and uniform. Though people are more likely to migrate to local towns and cities, and most stay within their birth country, we assume that the net effect on the global pattern is equivalent to random migration. For reproduction, we assume a uniform growth rate across all populations. Because we are most interested in global patterns, we ignore fine-scale differences across regions that are observed empirically. Along with death, migration and reproduction are the only means for changing population size.

More detailed descriptions of this kind of reaction-diffusion model are elsewhere [11], [32]; here we will describe its general properties. In the model, we take a uniform, square-celled lattice of size *L* ^2^ and begin at time *t* = 0 with some initial population *n*(0) in each cell (all units are arbitrary and can be scaled to represent the resolution of the model). There are three steps that occur from time *t* to *t*+1. In step one, we induce random, global migration by redistributing the population: each cell independently either increases to (1/*ρ*)*n* with probability *ρ* or decreases to 0 with probability 1−*ρ*. Step two represents urban sprawl: a fixed proportion *α* of each cell is distributed among its four nearest neighbors. (The lattice has periodic boundaries, so there are no edge effects.) In step three we increase the population of each cell by some small proportion *r* to represent reproduction. The model is then iterated to some time *T*>1000 to resolve any transient behavior. In this type of stochastic reaction-diffusion model without the reproduction term, the global population Σ*n*(*t*) naturally converges to zero as *t*→*∞* [11]; therefore, a small population correction is made if Σ*n*(*t*)<Σ*n*(0). On average, this only occurred in 4% of the time steps in each simulation. The parameters of the simulation are *L* = 256, *n*(0) = 10, *ρ* = 0.5, *α* = 0.25, *r* = 0.025, *T* = 1024.

**Figure 3:**
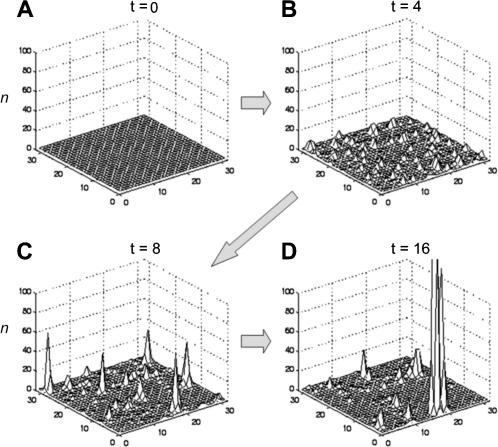
Development of the urban growth simulation, shown here on a small lattice of *L* = 32. a) At time *t* = 0 there is some population *n*(0) in each cell. b) Time *t* = 4. c) Time *t* = 8. d) By time *t* = 16, intermittent spikes of large population are clustered together in an otherwise sparsely-populated lattice.

By the end of each simulation, most cells have very small populations of 0≤*n*(*T*)<1. (See Figure 3 for an illustration of the model's evolution on a smaller lattice.) Intermittent spikes of very large population are scattered sparsely across the lattice. These high-population cells appear in clusters throughout the lattice since nearest neighbors exchange population. The population distribution of the lattice can be measured in several ways: 1) every cell *n*(*T*); 2) only “large” cells *n*(*T*)>1; 3) cluster populations, summed across all cells in a cluster; or 4) cluster area measured as the number of cells in a cluster. These correspond to different measurement criteria for real cities: all cities, large cities only (the tail of the distribution), metropolitan area population and metropolitan area extent.

## Results

### Empirical results

**Figure 4:**
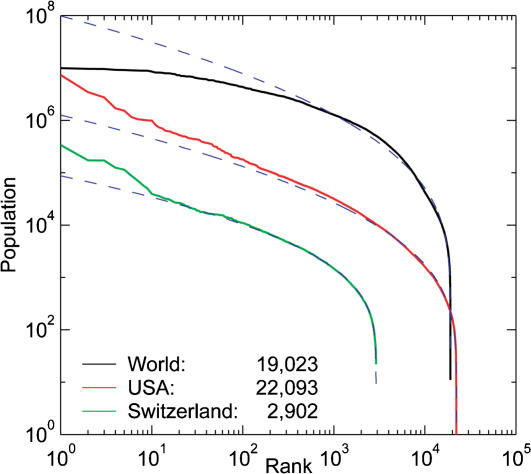
Rank-size distributions and lognormal fits (dashed blue lines) for all ‘counties’ (2 administrative levels below the nation) of the world, all populated places of the USA, and all municipalities of Switzerland. All three samples are fit well by a lognormal over four orders of magnitude in population, particularly in the bodies of the distributions. Key indicates sample sizes.

**Figure 5:**
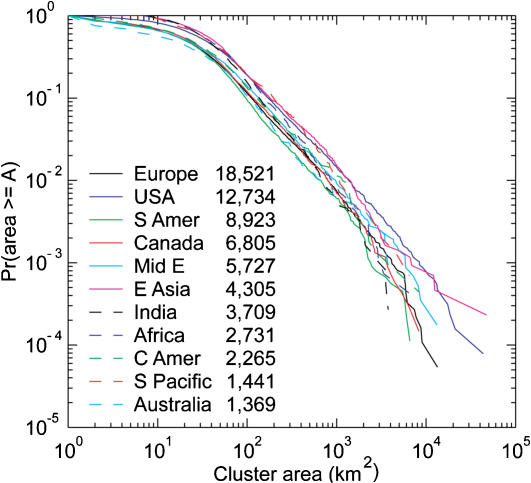
Cumulative probability distributions of nighttime light clusters for continental and sub-continental regions. Key indicates number of clusters in each region (total *N* = 68,530). The body of each distribution is fit well by a lognormal (not shown). The tails (largest clusters) are fit well by power laws (not shown). Slopes of the scaling regions are all significantly different from the Zipf's law expectation of −1.

For both the census data (Figure 4) and the night light clusters (Figure 5), the tails of all distributions (e.g., light cluster areas exceeding 50 km^2^) appear to approximate power laws (using maximum likelihood estimation). However, slopes of power laws (fitted over the large cities only) are almost all significantly different from each other and lie between −0.729 and −0.888, far from the theoretically expected Zipf exponent of −1.

More remarkably, when all city sizes are considered, the bulk of the data are better fit by a lognormal distribution than by a power law. Figure 4 shows the rank-size distributions for the same locations as Figures 1 and 2, but this time including all settlements, which increases the sample size ten-fold for Swiss municipalities, 58-fold for World ‘counties’ (2 administrative levels below the nation), and 75-fold for US populated places. Maximum likelihood estimates of the lognormal parameters (*μ* and *σ*) for of the world (*μ* = 10.95, *σ* = 1.92, *r* ^2^ = 0.439, *p*<0.0001), all populated places of the USA (*μ* = 7.26, *σ* = 1.73, *r* ^2^ = 0.840, *p*<0.0001), and all municipalities of Switzerland (*μ* = 6.77, *σ* = 1.36, *r* ^2^ = 0.858, *p*<0.0001) fit the data very well over four orders of magnitude in population size, particularly in the bodies of the distributions.

Similar patterns occur in the nighttime light data. Figure 5 shows cumulative probability distributions (another way of plotting rank-size relationships) of nighttime light clusters for continental and sub-continental regions around the world. Sample sizes in each region range from 1,369 light clusters in Australia to 18,521 clusters in Europe. (Delineation of regions did not precisely follow political boundaries.) Clusters are defined with the four-cell neighborhood rule, such that illuminated cells are contiguous and therefore members of the same cluster only if they touch along one of their four sides. As with the population data, the body of each distribution is fit well by a lognormal (MLE parameters were all significant), and the tails (largest clusters) are fit well by power laws (fits not shown in the figure) whose slopes are between −0.729 and −0.888, all significantly different from the Zipf's law expectation of −1.

All data sets have similarly-shaped lognormal distributions despite large differences in socioeconomic factors, settlement history, region size, and measurement criteria. The bodies of the distributions (which contain the bulk of the data) are clearly lognormal. Interestingly, for the global population data, the lognormal overestimates the population of the largest cities (i.e., the lognormal tail is too heavy, Fig. 4), whereas for more regional data, lognormal estimates are always too low for the largest cities (i.e., the tail is too light). Even though developed regions have more cities and larger urban agglomerations, the character of the distributions is strikingly similar across regions (Fig. 5). Table 1 summarizes the lognormal parameter estimates for the population data.

**Table 1:** Lognormal parameter estimates for city size distributions^a^

Region	n	*μ*	*σ*	*r^2^*	*p*
World counties	19,023	10.95	1.92	0.439	<0.0001
USA populated places	22,093	7.26	1.73	0.840	<0.0001
Swiss municipalities	2,902	6.77	1.36	0.858	<0.0001
Simulation, all cells	62,983	2.90	3.15	0.660	<0.00001

a In the lognormal model, *ln*(Size) has the normal distribution with mean *μ* and standard deviation *σ*. n: sample size. Parameters fitted using Maximum Likelihood Estimates.

### Model results

**Figure 6:**
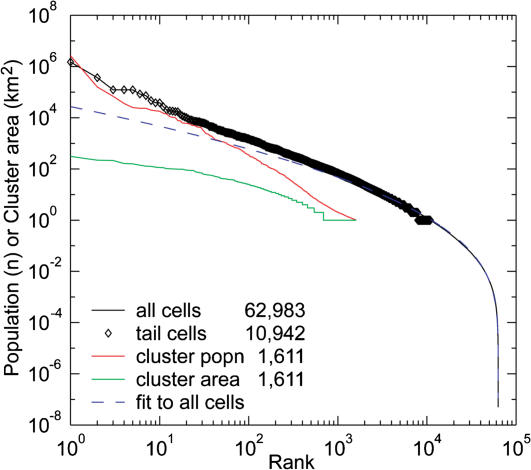
Rank-size distributions of cities from the simulation. The lognormal fit (dashed line) is statistically indistinguishable from the curve for all cells. The three tail distributions are fit well by power laws (fits not shown).

The model generates lognormal distributions (fitted using maximum likelihood estimation) of city sizes that mimic power laws for the largest cities and are very similar to those found empirically (Figure 6). The lognormal fit is statistically indistinguishable from the distribution of all cells (*μ* = −2.90, *σ* = 3.15, *r* ^2^ = 0.660, *p*<0.00001), though it does deviate from the data for the largest cells. As expected, the slopes and sample sizes of the tail varies depending on the measurement criteria. But all three tail distributions are fit well by power laws: tail cells with *n*(*T*)>1 (*P* = 7.47×10^6^ *R* ^−1.702^, *s.e.* = 0.002, *r* ^2^ = 0.992, *p*<0.00001), cluster populations (*P* = 3.97×10^6^ *R* ^−2.079^, *s.e.* = 0.005, *r* ^2^ = 0.963, *p*<0.00001), and cluster areas (*A* = 3.99×10^4^ *R* ^−1.169^, *s.e.* = 0.007, *r* ^2^ = 0.525, *p*<0.00001).

These results are relatively robust for 0.41<*ρ*<0.592, 0.1<*α*<0.35, and *r*<0.5. The limits of these parameters are mechanically related to the neighborhood rule and the geometry of the lattice that are used, and little demographic meaning can be ascribed to them. For example, for any lattice where *ρ*≥0.592, a single cluster forms that spans the whole lattice. The value of 0.592 is well known from percolation theory to be a critical value for the connectivity of square cells with a four-cell neighborhood [33]. Analytic solutions to such a lattice (though without reproduction, such that *r* = 0) have been worked out [11], [32] and show, for example, that the scaling of cluster populations breaks down when *ρ* varies across the lattice. Parameters for the lognormal fit are also in Table 1.

## Discussion

We have shown that for all regions of the world, most human settlements are distributed lognormally, whether by population or by area. Even though we have used data sets with very different measurement criteria, our analysis shows the same pattern: a lognormal distribution with the largest cities approaching a power law. Eeckhout [34] analyze US census data and also find that the distribution is lognormal when all cities are included in the analysis. This appears to be a general feature of human settlement patterns that is robust to changes in measurement criteria, socioeconomic factors, and settlement history. We have also shown that the largest cities deviate from the lognormal and approach a power law, that the scaling exponent is significantly less than−1, and that there is substantial systematic deviation from the power law fit. This supports Gabaix&Ioannides' [21] conclusion that exponents are lower for urban agglomerations than for politically defined cities. While night light data suggest that all regions are converging onto a power law, lognormals are known to mimic power laws over a broad range of values if the variance is large [35], [36]. The type of fit is more than a semantic argument; lognormal distributions are indicative of probabilistic, multiplicative processes quite different from those suggested by power laws.

Results from the model not only support the empirical findings, but demonstrate that fundamental demographic (i.e., ecological) behaviors can account for this universal pattern. Although the model is extremely simple, the multiplicative process of population aggregation suffices to generate a lognormal distribution of cell population sizes with the largest cells approaching a power law. We expect quantitative measures of real city distributions may differ from our results somewhat due to spatial heterogeneity of the substrate (which would result in spatial variation of *n*(0), *ρ*, *α* and *r*), non-random migration, finite size effects, and other factors not accounted for in this model.

As these patterns appear to be global—insensitive to regional history, topography, climate, and socioeconomic factors—it is likely that human populations are constrained by some fundamental laws related to the flow and distribution of resources within and among cities. Because sociological and economic processes ultimately serve the ecological needs of humans for survival and reproduction, patterns of urban distribution should be explicable in ecological terms [37], [38]. Research on fractal scaling in river basins [39] and allometric scaling in organisms [40] illustrates how energy minimization principles and conservation laws can govern the structure and function of complex natural systems [41]. This body of theory is based on the premise that hierarchical branching networks efficiently distribute energy and materials through landscapes (in the case of river networks) and organisms (in the case of vascular networks) across orders of magnitude in scale. We hypothesize that the distribution of resources within and among cities should be governed by principles similar to those that appear to underlie the physiology of organisms and structures of ecosystems [40]–[44].

There is evidence that human populations adhere to these same principles. For example, human reproductive output is related to per capita power consumption by the same scaling laws that describe reproductive effort for all other mammal species—even though modern humans consume most of their energy in the form of fossil fuels rather than food [45]. Bettencourt [46] shows how scaling principles may provide a framework for a quantitative understanding of city growth. There is also reason to believe that urban systems should develop highly effective network structures: whereas natural ecosystems flux between 1,000 and 10,000 Kcal m^−2^ yr^−1^, industrialized cities flux between 100,000 and 300,000 Kcal m^−2^ yr^−1^ [47]. This 10-fold increase in the energy throughput of urban areas ought to be both a product and driver of a highly developed network structure [41].

Some urban network structure is easily visible, for example in the hierarchical branching of road networks from highways to urban arteries and residential streets. Road networks determine the rate of flow of people and goods in cities, in much the same way that the cardiovascular system determines the rate of oxygen delivery to cells [48]. Similar to the way that body mass influences circulation times in organisms, city area and population size are key determinants of transportation time through urban road networks. Thus, in addition to the ecological processes of birth, death and migration, city size distributions may also reflect fundamental properties of urban networks. Interestingly, body size distributions have also been characterized as both lognormal [49] (although sometimes skewed lognormal [50]) and power law [42]. Indeed, common distributions may result from very general processes in natural, economic and engineered systems [51].

Cities are elements in what has become a global network that distributes people, food, energy, materials, wealth and information. While city networks probably differ from river basins and cardiovascular systems in fundamental ways, similar principles likely apply to their dynamics.

## Supporting Information

Figure S1 Raw data for population sizes of the US, Switzerland, and the world (used in Figures 1 and figure 3).
(1.10 MB XLS)
